# Identification of the U-box gene family in peach (*Prunus persica*) and functional analysis of *PpPUB20* in response to salt stress

**DOI:** 10.3389/fgene.2025.1549981

**Published:** 2025-06-12

**Authors:** Huiwen Zhang, Ruxuan Guo, Xiaoshuang Zhang, Chongyu Zhao, Junkai Wu, Xiao Xiao, Yanhong Shen, Chunsheng Liu, Gang Li, Kai Su, Kun Xiao, Chenguang Zhang, Libin Zhang

**Affiliations:** Hebei Key Laboratory of Horticultural Germplasm Excavation and Innovative Utilization, Hebei Higher Institute Application Technology Research and Development Center of Horticultural Plant Biological Breeding, College of Horticulture Technology, Hebei Normal University of Science and Technology, Changli, China

**Keywords:** peach, U-box ubiquitin ligase, gene function, low-temperature stress, salt stress

## Abstract

**Background:**

With the rising proportion of saline soils in the global irrigated soil area, improving salt stress tolerance in peach is of great significance and value for the development of peach industry. Plant U-box proteins (PUBs) are widely involved in various stress response processes. In this study, genome-wide identification and analysis of PUBs genes in cultivated peach were carried out, and the expression profiles of peach PUB genes in different tissues of peach as well as their responses under salt stress were also investigated.

**Methods:**

The genome-wide identification of PUBs genes in cultivated peach was analysed by gene localisation, gene structure and evolutionary analysis. Subsequently, the expression profiles of PpPUB genes in different tissues of peach and the changes in the relative expression of peach PUB genes under ABA, GA3, IAA, 6-BA treatments, low-temperature stress and salt stress were investigated.

**Results and discussion:**

In this study, 51 U-box protein genes (PUB) were identified in the cultivated peach “*SJZX*” and divided into six groups. Most of the PpPUB were predicted to be located in the nucleus and chloroplasts. Promoter analyses indicated that most members may be associated with lightresponsive processes. Expression analysis based on RT-qPCR showed that most PUB members in peach were highly expressed in a certain tissues or organs. Based on the results of RT-qPCR expression analysis of 18 representative PpPUB after abiotic stress and hormone induction, all detected genes except for PpPUB19 were induced by salt stress, and *PpPUB3/20/23/49* were induced by low temperature. Multiple genes were induced or repressed by exogenous hormone treatments. Furthermore, Arabidopsis seedlings heterologously overexpressing *PpPUB20* exhibited greater salt tolerance than wild-type seedlings under the same salt stress conditions. These findings provide comprehensive information on the *PpPUB* family and identify *PpPUB* members that may be involved in the regulation of hormones and salt stress. Therefore, this study enhances the understanding of potential role of *PpPUB* in stress adaptation in peach, thereby establishing a foundation for subsequent functional investigations and applications in stress-resistant crop breeding.

## 1 Introduction

Ubiquitination refers to the process of attaching a ubiquitin molecule to a specific lysine residue on a target protein via the action of ubiquitin ligases. The most common form of ubiquitination is polyubiquitination, which involves formation of a polyubiquitin chain on a protein. This polyubiquitinated protein is susceptible to proteasomal recognition and degradation, thereby inhibiting or enhancing various physiological and biochemical reactions (Davis et al., 2021). In some cases, mono-ubiquitination can occur when the target protein is connected to a single ubiquitin molecule. Although mono-ubiquitinated proteins may not be recognized and degraded by proteasomes, they can still change their localization in cells by binding to other proteins with ubiquitin-binding domains; therefore, their function may also change (Emmerich and Cohen, 2015; Ma et al., 2020).

Ubiquitinated E3 ligase is a component of the ubiquitin/26S proteasome system (UPS), which is involved in regulating growth and development (Dye and Schulman, 2007; Davis et al., 2021) and responding to biotic and abiotic stresses (Davis et al., 2021) in plants. The UPS comprises three key enzymes: ubiquitin-activating enzymes (E1), ubiquitin-conjugating enzymes (E2), and ubiquitin ligases (E3) (Dye and Schulman, 2007). Ubiquitin is activated by E1 and adenosine triphosphate to form an E1-ubiquitin intermediate that interacts with E2. Activated ubiquitin is then transferred to the cysteine activation site of E2 to form an E2-ubiquitin intermediate (Ramadan et al., 2015). Finally, E3 interacts with E2-ubiquitin and its target protein and transfer the ubiquitin to the target protein (Iconomou and Saunders, 2016). According to the E3 type, this step can occur differently. Therefore, E3 determines the specificity of the UPS (Ramadan et al., 2015). E3 ubiquitin ligases exist in various types and can be classified based on their structure (single- or multi-subunit) and the mechanism of ubiquitin transfer. Their conserved domains are crucial for ubiquitination. Based on their mode of action and the characteristics and structural composition of the target protein, E3 ligases are mainly classified into four categories: Really Interesting New Gene (RING), U-box, Homologous to E6-associated protein Carboxy1 Terminus, and Cullin-RING Ligase (Dove et al., 2016).

Plant U-box type E3 ubiquitin ligases (PUBs) not only play a crucial role in protein degradation processes in plants but also participate in various cellular and biological tolerance processes, such as plant hormone regulation and biotic and abiotic stress responses (Trujillo, 2018; Trenner et al., 2022). In recent years, considerable research has focused on the role of PUBs in plant stress responses (Mao et al., 2022). *Arabidopsis PUB22* and *PUB23* act synergistically as homologous genes on cytoplasmic *RPN12a* to regulate the drought signaling pathway. However, they are highly similar to wild-type (WT) plants in terms of abscisic acid (ABA)-mediated stomatal movement. Later, it was found that *AtPUB18* and *AtPUB19* negatively regulated ABA-mediated drought stress responses independent of *AtPUB22* and *AtPUB23*. Importantly, the *AtPUB18-2 AtPUB19-3 AtPUB22 AtPUB23* quadruple mutant exhibited a stronger tolerance to drought stress (Seo et al., 2012). In addition, *AtPUB11* negatively regulated the ABA-mediated drought response (Chen et al., 2021). The overexpression of *TaPUB1* upregulates the expression of ion channel-related genes and positively regulates salt-stress tolerance in wheat (Wang et al., 2020b). In *Marchantia polymorpha*, the *MpPUB9* encodes a U-box E3 ubiquitin ligase that interacts with the exocyst protein MpEXO70.1 to regulate protein turnover and salt stress response (Lim et al., 2024). Additionally, in wheat (*Triticum aestivum* L.), the U-box E3 ligase gene *TaPUB15* enhances salt tolerance. Overexpression of *TaPUB15* in transgenic rice and *Arabidopsis* resulted in improved salt tolerance, increased root growth, and better ion homeostasis under salt stress. The expression of salt stress-related genes, including those involved in cell wall organization and ion transport, was also significantly upregulated in these transgenic plants (Li et al., 2021). *OsPUB7* negatively regulates salt stress in rice (*Oryza sativa* L.) (Kim et al., 2023). *CaPUB1* is not only a negative regulator of the drought stress-response in transgenic rice but also a positive regulator of the cold-stress response (Min et al., 2016). *Vitis pseudoreticulata VpPUB24* interacts with *VpICE1* and positively regulates cold tolerance (Yao et al., 2017). In addition, the OST1-PUB25/26 module regulates the cold-stress response in *Arabidopsis* by controlling the steady state of the negative regulator MYB15 (Wang et al., 2019b). Wheat *TaPUB1* improves heavy-metal tolerance by regulating the expression of antioxidant-related genes and antioxidant enzyme activity under Cd stress (Zhang et al., 2021).

Peaches (*Prunus persica* L.) have been cultivated in China for 3,000 years. As of 2020, the cultivation area of peach in China is approximately 780,000 ha, and the yield is more than 15 million tons. The top three provinces of peach cultivation area and yield in China are Shandong, Hebei, and Henan provinces (Xu and chen, 2023). By 2020, salinized soil accounted for more than 20% of the global irrigated soil area, and it is expected to expand to more than 50% by 2050 in a worst-case scenario (Singh, 2021). Referring to data from the Open Geospatial Laboratory (https://www.osgeo.cn/), as of 2016, Hebei, Shandong, and Henan provinces encompass a total of approximately 2.13 million ha of saline-alkali land, accounting for about 10% of the total cultivated land area 1.69 million ha of this saline-alkali land was in Hebei province, accounting for 9% of the total area of the province. Soil salinization, as one of the most serious abiotic stresses affecting plants, has attracted increasing attention. In-depth studies on plant salt tolerance have been conducted for many plant species (Li et al., 2024; Wang et al., 2024; Yuan et al., 2024). *PpTGA9*, which is highly homologous to the *Arabidopsis* transcription factor TGA9, can improve plant salt tolerance by interacting with *PpATP1* (Wu et al., 2023). However, studies on the resistance of peach to salt stress are limited.

In this study, we conducted a genome-wide identification and analysis of PUB gene family in cultivated peaches using gene mapping and structure analysis, evolutionary analysis, co-expression, and protein-protein interaction analysis. The stress response of the peach PUB gene was explored over a short period of time (within 4 h) under salt and low temperature stresses (Liang et al., 2022; Li et al., 2023). We then studied the expression profiles of *PpPUB* in different peach tissues and their responses to ABA, gibberellic acid (GA3), 3-indoleacetic acid (IAA), and 6-benzyladenine (6-BA) treatments. The results of this study will help us understand the role of PUB genes in peach growth, development, and stress resistance and enrich our knowledge of the response regulatory network.

## 2 Materials and methods

### 2.1 Identification of peach PUB family members

Some known *Arabidopsis* PUB mRNA sequences were download from The Arabidopsis Information Resource (TAIR) database (https://www.arabidopsis.org/) as a template. Then, the peach mRNA sequences were searched in the Prunus persica Zhongyoutao14v1.0 genome database from the Genome Database for Rosaceae (GDR) (https://www.rosaceae.org) using the Blastn algorithm to validate the candidate genes. The obtained sequence was uploaded onto the HMMER 3.0 software, and the hidden Markov model (HMM) map of the U-box domain (PF04564.15) was used to confirm whether the sequence contained the U-box domain. The confirmed peach PUB family member mRNA sequences were translated into protein sequences using the SnapGene Viewer software (www.snapgene.com). The physicochemical properties of the PUB proteins were predicted and analyzed using ProtParam (https://www.expasy.org/resources/protparam; ExPASy, Geneva, Switzerland). Finally, the subcellular localization of the peach PUB proteins was predicted and analyzed by using WoLF PSORT (https://wolfpsort.hgc.jp/; Tokyo, Japan).

### 2.2 Evolutionary analysis of the peach PUB family

The PUB protein sequence of *Arabidopsis thaliana* was downloaded from the TAIR database (https://www.arabidopsis.org/). Clustal W was used to perform multiple sequence alignment of the amino acid sequences of PUB proteins in Arabidopsis and peach. The evolutionary relationship was analyzed using the Molecular Evolutionary Genetics Analysis (MEGA) 11.0.13 software, and the phylogenetic tree was constructed by neighbor-joining (NJ). The verification parameter Bootstrap was set to 500 replicates, and the iTOL online program (https://itol.embl.de/) and Adobe Illustrator software (https://www.adobe.com/products/illustrator.html) were used for beautification.

### 2.3 Comprehensive analysis of peach family PUB genes

Gene Structure Display Server v.2.0 (http://gsds.cbi.pku.edu.cn/index.php) was used to compare the peach PUB gene coding sequences (CDSs) with the corresponding genomic DNA sequence, and their gene structure was analyzed and visualized. The MEME online program (http://meme-suite.org/tools/meme) was used to identify the conserved motifs of PpPUB protein. The number of motifs was set to 5, and the rest was the default. The 2,000 bp gene sequences upstream of the initiation codon of peach PUB family genes were extracted by Sequence Retrieval tools in GDR, and uploaded to PlantCARE (http://bioinformatics.psb.ugent.be/webtools/plantcare/html/) to predict and analyze the cis-acting elements in the promoter region, and the number distribution heat map of cis-acting elements was drawn using the online program (http://www.bioinformatics.com.cn/plot_basic_gopathway_enrichment_bubbleplot_081). TBtools-Ⅱ was used to analyze the collinearity of peach PUB family genes.

### 2.4 Plant materials and treatments

Three test peach tree materials were sourced from the Horticultural Experiment Station of Horticultural Science and Technology College of Hebei Normal University of Science and Technology. *A. thaliana* experiments involved the wild-type (WT) ecotype Columbia-0 (Col-0). In the summer of 2023, the fibrous roots, 1-year-old branches, mature leaves, buds and fruits of “SJZX”, which grew well under natural conditions, were taken as different tissue materials for gene expression analysis.

The 1-year-old potted “SJZX” with similar and good growth status was used as the stress treatment material. The potted “SJZX” was placed in the horticultural experimental station of Hebei Normal University of Science and Technology for normal cultivation and management, and then the fresh leaves of peach trees were treated with exogenous ABA, GA3, IAA, 6-BA, and low temperature. At the same time, salt treatment was carried out on the two-year-old peach.

Plant growth regulator treatment: 50 mg L^−1^ ABA, 700 mg L^−1^ GA^3^, 300 mg L^−1^ IAA and 300 mg L^−1^ 6-BA solutions were sprayed on peach leaves. Low temperature treatment: 0°C culture. The above three treatments were sampled at 0 h, 1 h, 2 h and 4 h, respectively. Salt treatment: the roots of peach trees were watered with 200 mM NaCl solution and sampled at 0 h, 3 h, 6 h, 12 h and 24 h respectively. The peach trees cultured normally in the same period were used as controls.

The above treatments were repeated three times. The samples were frozen in liquid nitrogen and stored in a refrigerator at −80°C for later use.

### 2.5 RNA isolation and cDNA synthesis

Total RNA was extracted from the samples using a polysaccharide polyphenol plant total RNA extraction kit (Tiangen, Beijing, China). The integrity and purity were examined via 1% agarose gel electrophoresis, and the concentration was examined using an ultraviolet spectrophotometer (manufacturer, City, Abb. State, Country). Total RNA was reverse transcribed into cDNA using the FastKing cDNA first-strand synthesis kit (Tiangen, Beijing, China) for quantitative reverse transcription polymerase chain reaction (RT-qPCR) detection.

### 2.6 Expression analysis of PUB gene in peach

Expression analysis based on RT-qPCR was perfomed using SuperReal fluorescence quantitative premix reagent enhanced version (SYBR Green) (Tiangen, Beijing, China) on CFX96 RT-qPCR system (Bio-Rad, Hercules, CA, United States), and α-tubilin (TUA) was selected as the internal reference gene for ABA, GA3, IAA, 6-BA, cold stress and expression analysis of related genes in different tissues. Primer Premier 6 software was used to design specific primers for RT-qPCR amplification (Supplementary Table S1). Each experiment was repeated three times. Fluorescence was recorded at the end of each cycle of annealing steps. The relative expression level of the *PpPUB* gene was calculated using the 2 ^−∆∆Ct method (Livak and Schmittgen, 2001). The original expression data were normalized. All data were expressed as mean ± standard deviation (SD) of three independent biological replicates.

### 2.7 Transformation of *Arabidopsis thaliana* and identification of transgenic plants

*Arabidopsis thaliana* ecotype Columbia Col-0 plants (WT) were transformed with heterologous overexpression of *PpPUB20* using the flower bud dipping method (Wiktorek-Smagur et al., 2009). T0 seeds were grown in Murashige and Skoog (MS) solid medium and 25 mg L^−1^ kanamycin to screen for positive strains. Three Arabidopsis strains (OE-1, OE-2, and OE-3) of OE-PpPUB20 were selected and T3 pure seeds plants were cultured for salt tolerance test.

### 2.8 Salt tolerance evaluation of transgenic lines

To verify the salt tolerance of *PpPUB20* transgenic lines, the seeds of *PpPUB20* transgenic lines and WT (control) were cultured in MS solid medium containing 0, 75 and 100 mM NaCl. The growth of seeds after germination was observed, and the root length of 10-day-old seedlings was measured.

### 2.9 Plants materials

Te collection and cultivation of Prunus persica “Shiji Zhixing” plants in the presented study strictly followed the guidelines and regulations at the locality and study site(s). Morphological features were analyzed as available to ensure accurate identifcation of the plants as “SJZX” and to diferentiate them from others (Song et al., 2022). ADJ plant material was procured from a residential setting only afer approved permission from the municipal council for the use of the Horticultural Experiment Station of Horticultural Science and Technology College of Hebei Normal University of Science and Technology. The identification of improved varieties of “SJZX” plant was carried out by Hebei Forest Tree Approval Committee, and the identification number was JIS-SV-PP-005-2020.

### 2.10 Statistical analysis

The data were analyzed using IBM SPSS statistical software (version 26.0) (https://www.ibm.com/cn-zh/spss). One-way analysis of variance was used to calculate the significant differences between groups by least significant difference (LSD) multiple comparison method, and the Tukey’s test was used to test differences among sample means for significance. P value ≤0.05 was considered statistically significant. Finally, the heat map and histogram were drawn using the online program (http://www.bioinformatics.com.cn/plot_basic_gopathway_enrichment_bubbleplot_081) and Prism 5 software (https://www.graphpad.com/support/prism-5-updates/).

## 3 Results

### 3.1 Genome-wide identification and phylogenetic relationship of *PpPUB*

**FIGURE 1:**
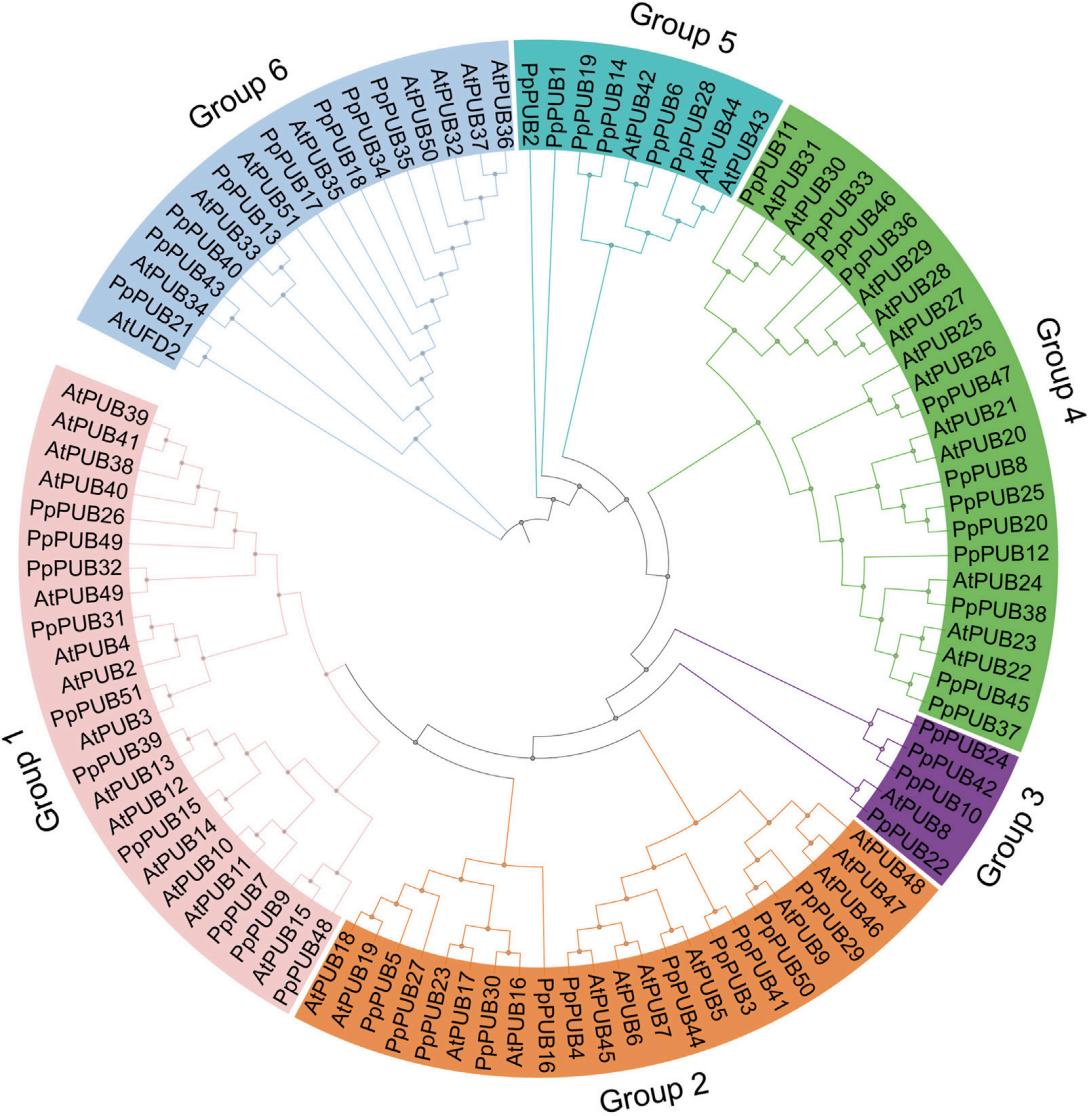
Phylogenetic relationship of PUB members in peach. Clustal W was used to perform multiple sequence alignment of the amino acid sequences of PUB proteins in Arabidopsis and peach. A phylogenetic tree based on the alignment above was constructed by the neighbor-joining (NJ) method with 1,000 bootstrap replicates. The *PUB* gene family members are divided into six groups (Group 1–6). The genes in different groups are highlighted in six different colors, respectively.

To identify the U-box E3 ubiquitin ligase gene in peach, the PUB mRNA sequence of *A. thaliana* was used as a query for BLAST in the peach database. Simultaneously, HMMER 3.0 software was used to identify the peach U-box E3 ubiquitin ligase gene using the hidden Markov model map of the U-box domain (PF04564.15). Fifty-one U-box E3 ubiquitin ligase genes were found in the peach genome and named *PpPUB1-51* according to their gene ID (Table 1). To analyze the phylogenetic relationship of the *PpPUB*, a phylogenetic tree based on the *PpPUB* protein sequence alignment was constructed using the NJ method, with a bootstrap repeat number of 500. The 51 PpPUB proteins were divided into six subfamilies (1–6 groups) containing 10, 11, 4, 12, 6, and 8 members, respectively (Figure 1). The characteristics of their *PpPUB*, including the number of amino acids (AA), protein molecular weight (MW), subcellular localization, genomic location and protein isoelectric point (pI), were then described (Table 1). The number of amino acids per PpPUB protein sequence ranged from 275 residues (PpPUB49) to 2,109 residues (PpPUB23) (Table 1). The molecular weight of the PpPUB proteins ranged between 31.3 kDa (PpPUB49) and 171.9 kDa (PpPUB23), and the isoelectric point was between pH 4.95 (PpPUB23) and 9.37 (PpPUB37) (Table 1). Subcellular localization prediction showed that most of the PpPUB proteins (86%) were nuclear, chloroplast, and cell-wall proteins, whereas other members were related to the plasma membrane, endoplasmic reticulum, peroxisomes, mitochondria, and vacuoles (Table 1).

**TABLE 1:** Characteristics of the putative peach PUB genes.

Gene ID	Proposed name	The number of amino acids	MW (KDa)	Subcellular location	Chromosome location	pI
Pp05G006280.1	*PpPUB26*	485	52.95	cyto	G5:15118593-15120080	5.92
Pp08G022450.1	*PpPUB49*	275	31.30	mito	G8:18741344-18743772	5.34
Pp06G014020.1	*PpPUB32*	798	91.26	cyto	G6:22815793-22819707	7.79
Pp06G013560.1	*PpPUB31*	838	91.24	nucl	G6:23142643-23160973	5.68
Pp08G025140.1	*PpPUB51*	777	84.99	chlo	G8:20238177-20243339	6.66
Pp06G033580.1	*PpPUB39*	666	72.12	chlo	G6:5378118-5383949	5.46
Pp02G027720.1	*PpPUB15*	708	78.22	plas	G2:24504154-24507878	6.66
Pp01G039140.1	*PpPUB7*	636	69.45	E.R.	G1:31599956-31602907	8.65
Pp01G042180.1	*PpPUB9*	606	67.07	pero	G1:33599483-33602105	5.61
Pp08G021070.1	*PpPUB48*	647	71.68	nucl	G8:17989233-17992437	6.01
Pp01G032330.1	*PpPUB5*	662	73.20	chlo	G1:27215813-27217873	8.96
Pp05G006580.1	*PpPUB27*	686	75.96	plas	G5:14971254-14973389	8.65
Pp04G006290.1	*PpPUB23*	2,109	171.90	chlo	G4:3409160-3411310	4.95
Pp06G006820.1	*PpPUB30*	402	42.82	cyto	G6:27142467-27143675	9.22
Pp02G031820.1	*PpPUB16*	674	74.05	nucl	G2:26981874-26983946	6.32
Pp01G029050.1	*PpPUB4*	776	85.18	nucl	G1:24822443-24828667	5.49
Pp07G010910.1	*PpPUB44*	578	65.4	nucl	G7:16427577-16433919	6.37
Pp01G026950.1	*PpPUB3*	1,025	114.00	nucl	G1:23172340-23177171	6.69
Pp06G036040.1	*PpPUB41*	1,043	115.37	chlo	G6:3820266-3825241	6.29
Pp08G022470.1	*PpPUB50*	456	50.7	cyto	G8:18749458-18752049	8.47
Pp06G000930.1	*PpPUB29*	1,395	114.92	E.R.	G6:30480920-30483872	5.02
Pp04G002920.2	*PpPUB22*	380	41.62	nucl	G4:1536609-1540672	8.46
Pp01G043280.1	*PpPUB10*	1,482	164.55	chlo	G1:34229047-34236602	5.68
Pp07G004350.1	*PpPUB42*	1,340	148.27	chlo	G7:20005132-20011899	5.32
Pp04G030290.1	*PpPUB24*	286	32.25	chlo	G4:23290774-23291854	7.09
Pp06G025530.1	*PpPUB37*	365	40.38	cyto	G6:12117782-12118954	9.37
Pp07G016870.1	*PpPUB45*	779	87.22	nucl	G7:12536422-12546165	8.82
Pp06G025560.1	*PpPUB38*	411	45.99	cyto	G6:12095574-12096809	8.43
Pp02G017700.1	*PpPUB12*	405	46.13	nucl	G2:16706280-16707527	8.12
Pp03G021790.1	*PpPUB20*	447	49.89	chlo	G3:18705580-18706923	6.64
Pp05G000900.1	*PpPUB25*	416	46.75	nucl	G5:18120696-18121976	5.93
Pp01G040470.1	*PpPUB8*	420	46.27	plas	G1:32438898-32440160	7.50
Pp08G016400.1	*PpPUB47*	369	40.01	cyto	G8:15147594-15148844	5.72
Pp06G022760.1	*PpPUB36*	404	44.16	chlo	G6:14821983-14823257	6.41
Pp08G002940.1	*PpPUB46*	411	45.19	chlo	G8:2274748-2275983	7.97
Pp06G014230.1	*PpPUB33*	449	49.10	cyto	G6:22670307-22671656	6.28
Pp01G048450.1	*PpPUB11*	456	49.41	nucl	G1:37104942-37106312	7.00
Pp05G025010.1	*PpPUB28*	813	89.45	cyto	G5:940720-945332	5.36
Pp01G036550.1	*PpPUB6*	1,034	115.92	cyto	G1:29979574-29983313	5.72
Pp02G026180.1	*PpPUB14*	1,008	111.72	chlo	G2:23438252-23444928	5.57
Pp02G037410.1	*PpPUB19*	1,008	111.75	chlo	G2:29989498-29994138	5.54
Pp01G002400.1	*PpPUB1*	1,025	114.00	nucl	G1:1774064-1779014	6.69
Pp01G004540.1	*PpPUB2*	776	85.18	chlo	G1:3353972-3361342	5.49
Pp06G015950.1	*PpPUB35*	819	93.69	nucl	G6:21184992-21189478	8.45
Pp06G015940.2	*PpPUB34*	798	91.26	cyto	G6:21191967-21196456	7.79
Pp02G035760.1	*PpPUB18*	489	55.10	nucl	G2:29123475-29128466	6.05
Pp02G033620.1	*PpPUB17*	808	90.38	nucl	G2:27931330-27936942	6.46
Pp02G024010.1	*PpPUB13*	867	97.55	nucl	G2:21995472-22001797	5.83
Pp06G033640.1	*PpPUB40*	642	71.92	nucl	G6:5327827-5333981	7.54
Pp07G006290.1	*PpPUB43*	688	77.69	cyto	G7:18953408-18957211	6.47
Pp03G031210.1	*PpPUB21*	702	76.55	chlo	G3:25083278-25089588	5.60

### 3.2 Conserved domain and exon-intron structure of *PpPUB*

**FIGURE 2:**
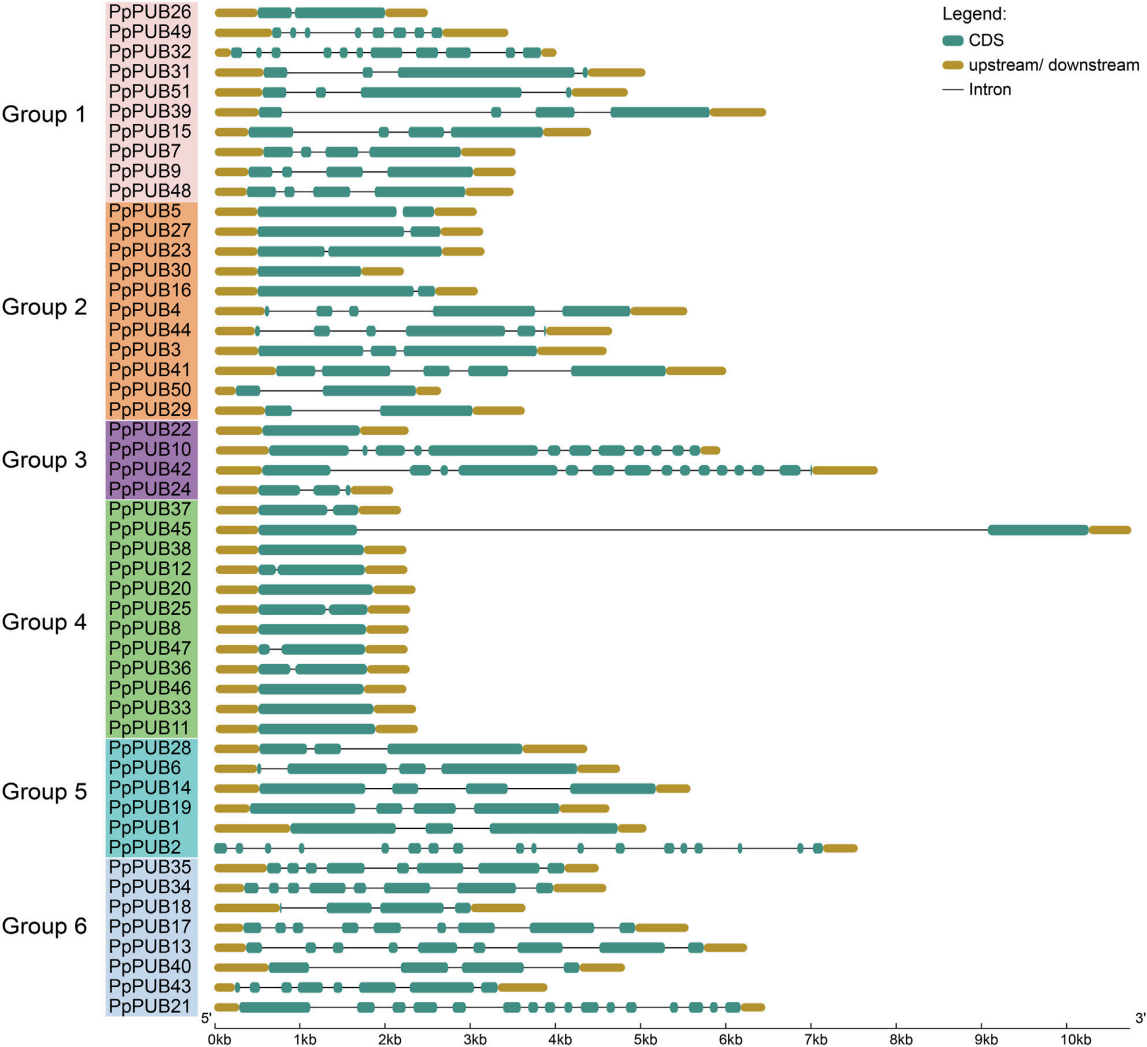
Gene structure predictions of PUB members in peach. The genomic and coding sequences of *PpPUB* genes were submitted to the Gene Structure Display Server to analyze the exon-intron structures of *PpPUB* genes.

To explore the exon-intron structure of *PpPUB*, its genome and coding sequences were submitted to GSDS 2.0. The number of exons in each *PpPUB* ranged from 1 to 18, and the exon-intron composition was relatively conserved within the same subfamily (Figure 2). The number of exons in the fourth group of genes was less than or equal to two, of which six genes (50%) contained only one exon; among the fifth group, one member contained 18 exons and 1 intron, and the others (83%) contained no more than four exons. Six members (63%) in group 6 contained eight or nine exons (Figure 2).

**FIGURE 3:**
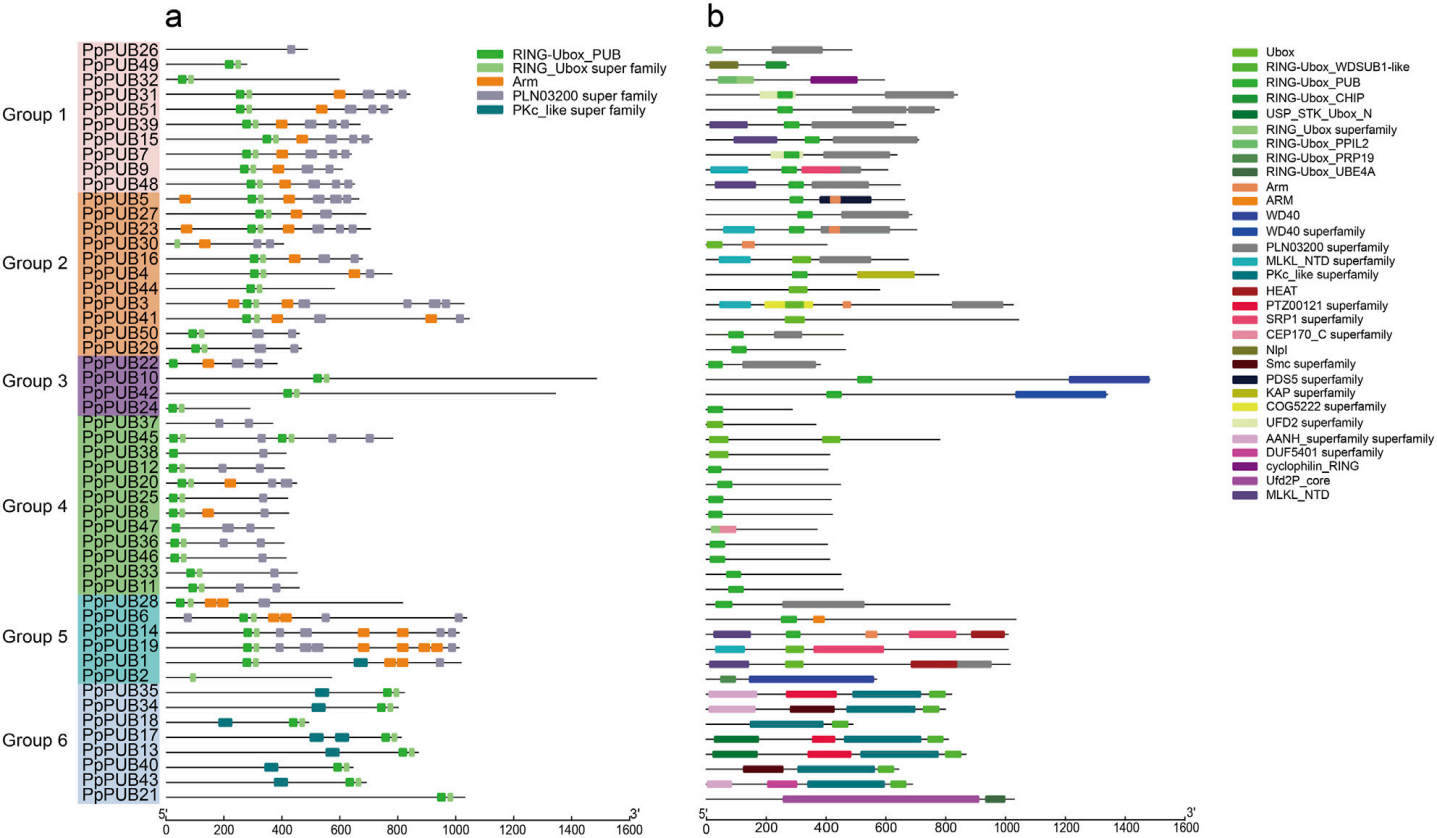
Conserved domains and conversed protein motifs of peach PUBs. **(a)** Phylogenetic relationship and distribution of 5 conserved motifs analyzed by MEME online tool in the 51 *PpPUB* proteins; **(b)** 51 *PpPUB* protein sequences were submitted to the NCBI to analyze the conserved domains of *PpPUB* proteins. The composition of conserved domains in the *PpPUB* protein sequences was visualized by IBM online tool. Thirty-one conserved domains marked in different colors are shown.

To further verify the classification results of the phylogenetic tree, we studied the conserved motifs and domains of PpPUB in peaches. Five motifs were estimated using the MEME online server (Figure 3a). Among them, RING_Ubox was present in all groups, indicating that it was highly conserved in all PpPUB proteins, and Armadillo/beta-catenin-like repeats (Arm) was present in 23 (45%) genes. Most members of the same subfamily contained the same motifs, indicating that they may have the same function. At the same time, the amino acid sequences wree submitted to Conserved Domain Search Service (https://www.ncbi.nlm.nih.gov/Structure/cdd/wrpsb.cgi). In the PpPUB protein family (Figure 3b), 31 conserved domains were identified: for example, U-box domain, Arm, WD40 repeats, cellulose synthase-interactive protein (PLN03200) domain, chromosome segregation ATPase (Smc) domain, karyopherin alpha (SRP1) domain, and HEAT repeats. The U-box domain was detected in all PpPUB members, which is consistent with the prediction results of the MEME online server. The other domains were distributed in different PpPUB protein subfamilies according to their evolutionary relationships (Figure 3b). Eleven members (92%) of the fourth group contained the U-box domain only. Different from the prediction results of the MEME online server, only six members had Arm domains, which may be because these proteins do not fold into a specific spatial conformation although they contain the necessary sequence to form the Arm domain. The PLN03200 domain was mainly present in members of groups 1, 2 and 3. Almost all members of group 6 (88%) contained the PKc_like domain (Figure 3b). In addition to some classical domains, we identified several special domains, including PTZ00121, Nlpl, COG5222 and DUF5401, which were uncommon in previous studies on the PUB gene family in Rosaceae plants (Wang et al., 2020a; 2021; Jiang et al., 2023).

### 3.3 Cis-regulatory elements in the *PpPUB* promoter

**FIGURE 4:**
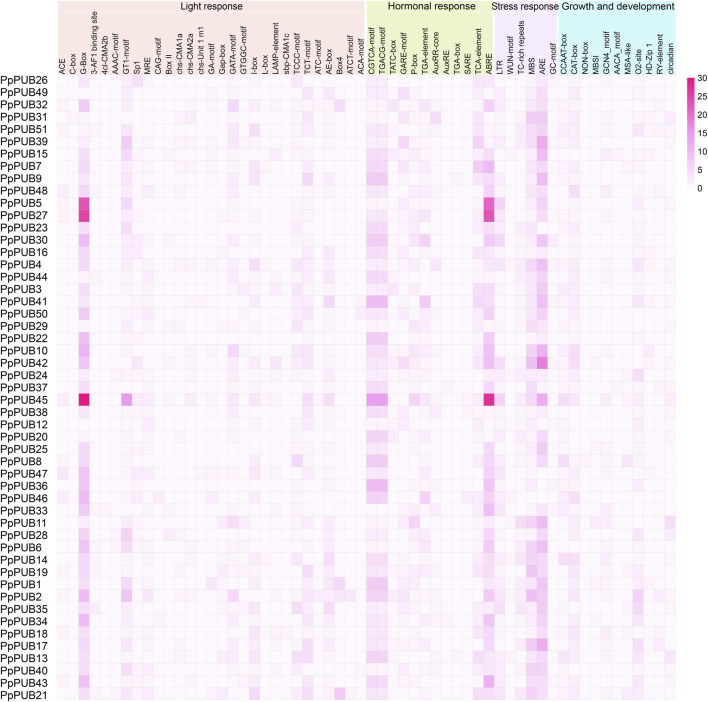
Heatmap of cis-regulatory element functional classification in the promoter of the *PpPUB* gene. Heatmap showing the number of individual cis-regulatory elements in the promoter of each *PpPUB* gene. Each row and column in the red grid indicate a single gene and cis-regulatory element, respectively, and their names are shown. Fifty-eight cis-regulatory elements are clustered into four categories based on corresponding biological processes, including growth and development process (growth and development), hormonal response, light response, and stress response.

As a transcription factor binding site, the cis-regulatory element upstream of the initiation codon is essential for the transcriptional regulation of protein-coding genes (Wittkopp and Kalay, 2012). To analyze the cis-regulatory elements in the *PpPUB* promoter, a 2.0 kb sequence upstream of the start codon of *PpPUB* was extracted and submitted to the PlantCARE database. We identified 58 cis-regulatory elements in the PpPUB promoter region (Figure 4; Supplementary Table S2). Based on the related biological processes, these cis-regulatory elements were divided into four groups: light response, including 29 elements; hormonal response, including 12 elements; stress response, including 6 components; and growth and development, including 11 components (Figure 4; Supplementary Table S2). Notably, of the 58 components, 897 (34%) and 913 (35%) belonged to the light- and phytohormone-response groups, respectively (Supplementary Table S2). Among the light-responsive elements, G-box was the most frequent member, accounting for 32% (Supplementary Table S2). In the plant hormone-response group, ABRE (28%) was involved in the ABA response and CGTCA-motif (19%) and TGACG-motif (19%) in the methyl jasmonate (MeJA) response accounted for a larger proportion (Supplementary Table S2). In the plant hormone-response group, the element ARE, which is essential for anaerobic induction, had the highest frequency of occurrence, accounting for 48% (Supplementary Table S2). These data suggest that *PpPUB* is likely involved in the response to light, ABA, MeJA, and oxygen.

### 3.4 Chromosomal localization and replication of *PpPUB*

**FIGURE 5:**
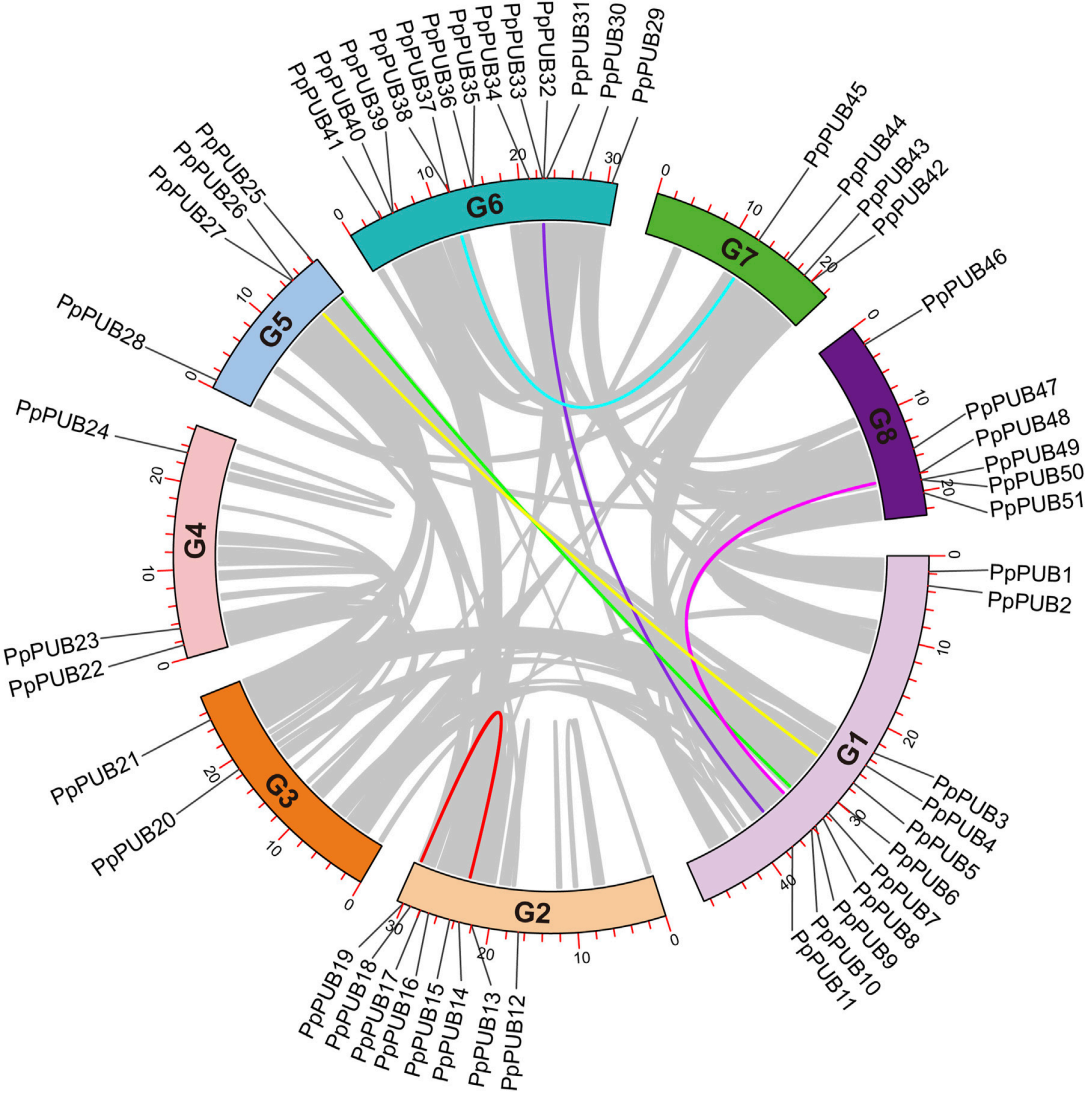
Collinearity analysis of *PUB* genes among peach. Eight chromosomes (G1–8) in peach are arranged in a circular pattern, and the location of 51 *PpPUB* genes is marked. Colored lines represent the homologous relationships of *PpPUB* genes.

To understand the chromosomal location of *PpPUB*, genomic information was extracted from the peach genome database and displayed on a genome map (Figure 5). Members of the *PpPUB* family were distributed on all eight chromosomes (G1–8). There were 11 *PpPUB* on G1, 8 on G2, 2 on G3, 3 on G4, 4 on G5, 13 on G6, 4 on G7, and 6 on G8 (Figure 5). Gene family formation is determined by gene replication events. Gene duplications (GDs) involve segmental duplication (SD) and tandem duplication (TD), which promote the expansion of gene families. To reveal the replication process of *PpPUB*, TBtools-Ⅱv1.12 software was used to analyze the collinearity of these genes, and the multi-locus genes located in adjacent regions or separated by uniform intergenic regions were identified. Sequences with repeat rates >90% and similarities >95% were designated as tandem repeats. The results showed that chromosome fragment duplication events occurred in six *PpPUB* pairs containing 13 genes and tandem duplication events occurred in another pair of *PpPUB* (Figure 5; Supplementary Table S3), indicating that fragment and tandem duplication events seemed to play a positive role in the expansion of the *PpPUB* family.

### 3.5 Temporal and spatial expression patterns of *PpPUB* in different tissues

**FIGURE 6:**
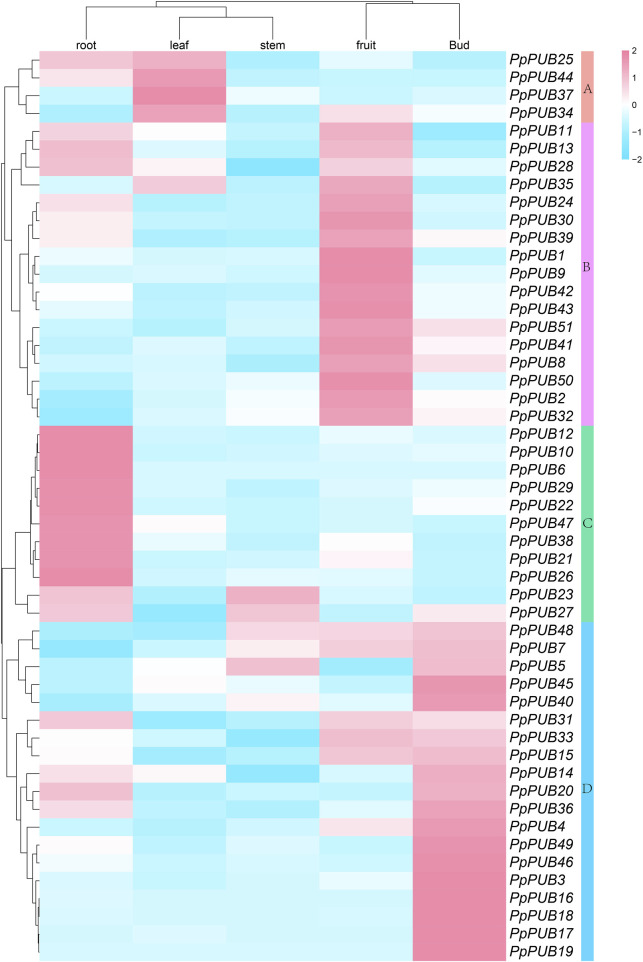
Expression analysis of *PpPUB* genes in five organs/tissues. The normalized expression data of *PpPUB* genes in five tissues, including root, stem, leaf, bud, and fruit, were obtained from the RT-qPCR data of peach. The expression levels of *PpPUB* genes were plotted in a log2-scaled heatmap. Each row and column in the color heatmap indicate a single gene and tissue, respectively, and their names are shown. Fifty-one *PpPUB* genes are hierarchically clustered into four groups (Groups A–D) according to their expression levels. Groups A–D are highlighted in five different colors, respectively.

Different tissue- and organ-specific gene expression patterns may indicate different biological functions. Understanding the expression of genes in different tissues is necessary to understand their biological functions in growth and development (Breschi et al., 2016). To confirm the tissue-specific expression of *PpPUB*, we performed tissue-specific analysis of 51 *PpPUB*, used RT-qPCR to analyze their relative expression levels in the roots, stems, leaves, buds, and fruits, and computed heat maps (Figure 6). As shown in Figure 6, 51 *PpPUB* were clustered into four groups (groups A–D) according to their transcriptional levels. Most genes were highly expressed in fixed tissues or organs, and the genes in groups B, C, and D were mainly expressed in the fruits, roots, and buds, respectively. The expression level of genes in group A was higher in the roots and buds than in the other three tissues. Most of the 51 *PpPUB* were highly expressed in the roots and leaves, and *PpPUB28* in group B showed low stem-specific expression. These results suggest that *PpPUB* is closely related to the growth and development of various tissues and organs.

### 3.6 Expression pattern of *PpPUB* in response to abiotic stress

**FIGURE 7:**
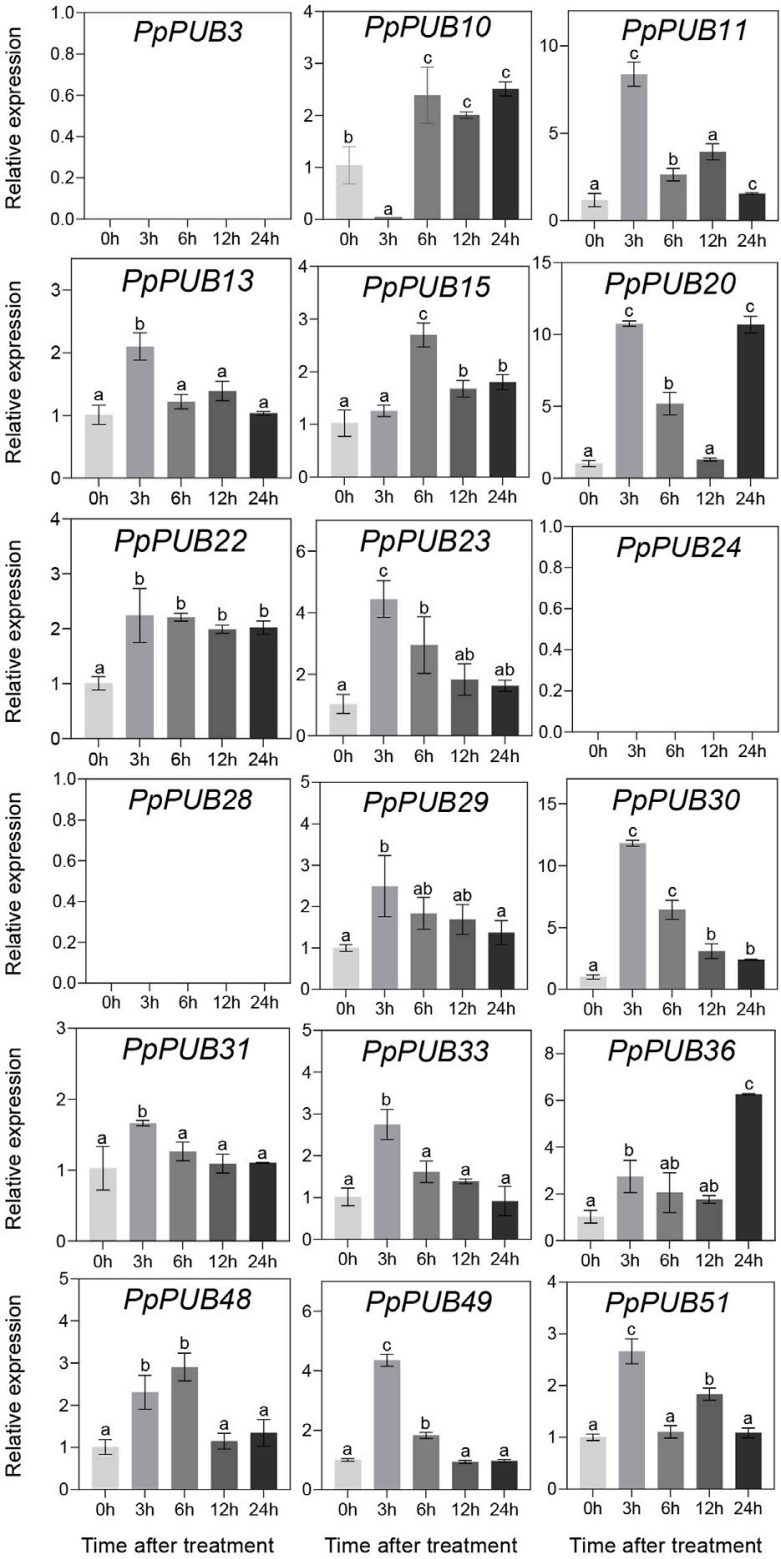
Relative expression levels of selected *PpPUB* genes under the salt-stress treatment. Total RNA was isolated from peach leaves after salt-stress treatment for 0 h, 3 h, 6 h, 12 h and 24 h, respectively. The extracted total RNA was then submitted to RT-qPCR. Error bars represent the means ± standard deviation (SD) of three independent experiments. Significant differences were determined by Duncan’s multiple range test (p < 0.005; one-way ANOVA). The lowercase letters above each bar indicates significant differences. Bars with the same lowercase letter represent no significant difference, and *vice versa*.

**FIGURE 8:**
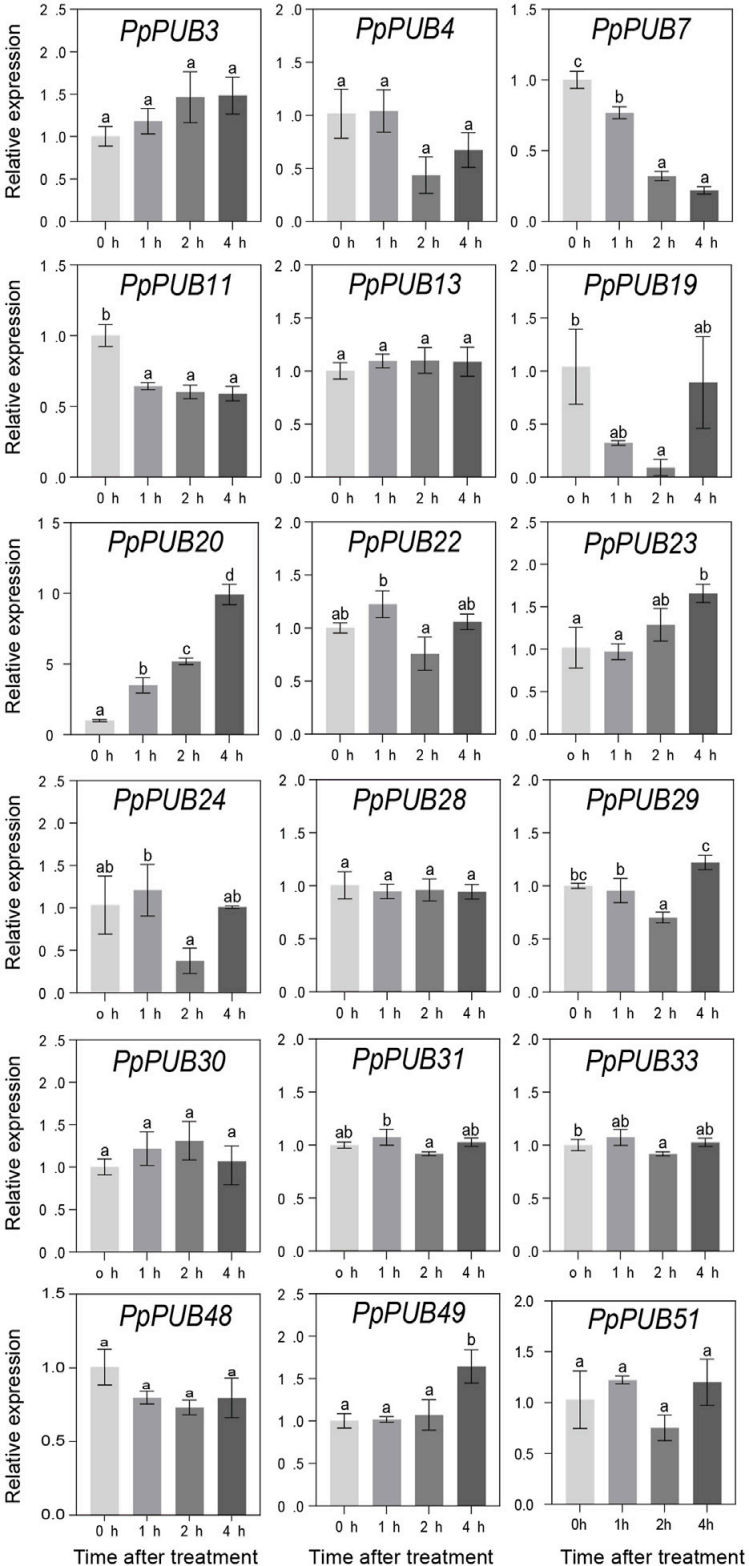
Relative expression levels of selected *PpPUB* genes under the low-temperature treatment. Total RNA was isolated from peach leaves after 0°C treatment for 0 h, 1 h, 2 h, and 4 h, respectively. The extracted total RNA was then submitted to quantitative real-time PCR. Error bars represent the means ± standard deviation (SD) of three independent experiments. Significant differences were determined by Duncan’s multiple range test (p < 0.005; one-way ANOVA). The lowercase letters above each bar indicates significant differences. Bars with the same lowercase letter represent no significant difference, and *vice versa*.

In *Arabidopsis* (Wang et al., 2019b), rice (Li et al., 2021), Antarctic moss (*Pohlia nutans*) (Wang et al., 2019a), and other plants, PUBs are involved in plant tolerance to abiotic stress. To better understand the role of *PpPUB* in response to low-temperature stress, at least one gene was randomly selected from each group of the *PpPUB* family as representative genes, and the relative expression levels of 18 representative genes under low temperature and salt stress conditions were analyzed via RTqPCR. The results showed that most of the assayed *PpPUB* responded to salt treatment (Figure 7). All genes responded quickly, and the expression increased or decreased rapidly after 3 h of treatment. The expression of 10 *PpPUB* (*PpPUB11/13/20/23/29/30/31/33/49/51*) peaked 3 h after salt-stress treatment (Figure 7). However, the expression of *PpPUB15* and *PpPUB48* gradually increased after treatment, peaked at 6 h, and decreased slightly after 12 h of treatment. Notably, *PpPUB3/24/28* were not expressed in peach seedlings. *PpPUB7* was not expressed after 3 h of treatment, and the expression increased significantly after 6 h. Other than that, the expression of *PpPUB11/20/30* increased significantly after salt treatment, indicating its role in the salt-stress response. In addition, the expression of 12 *PpPUB* (*PpPUB4/7/11/19/20/22/23/24/29/48/49/51*) changed significantly in response to low temperature (0 °C) treatment compared with those in the control group (Figure 8). Importantly, the transcription levels of four *PpPUB* (*PpPUB3/20/23/49*) showed an increasing trend within 4 h of low-temperature treatment. The expression of two *PpPUB* (*PpPUB7/11*) was significantly inhibited (Figure 8). In addition, the transcription levels of *PpPUB19* were significantly downregulated within 2 h of cold treatment and then upregulated to similar expression levels as before treatment after 4 h of cold treatment. Notably, *PpPUB20* was significantly upregulated under low-temperature conditions. These results suggest that *PpPUB* may be involved in the response to abiotic stress.

### 3.7 Expression pattern of *PpPUB* under hormone treatment

**FIGURE 9:**
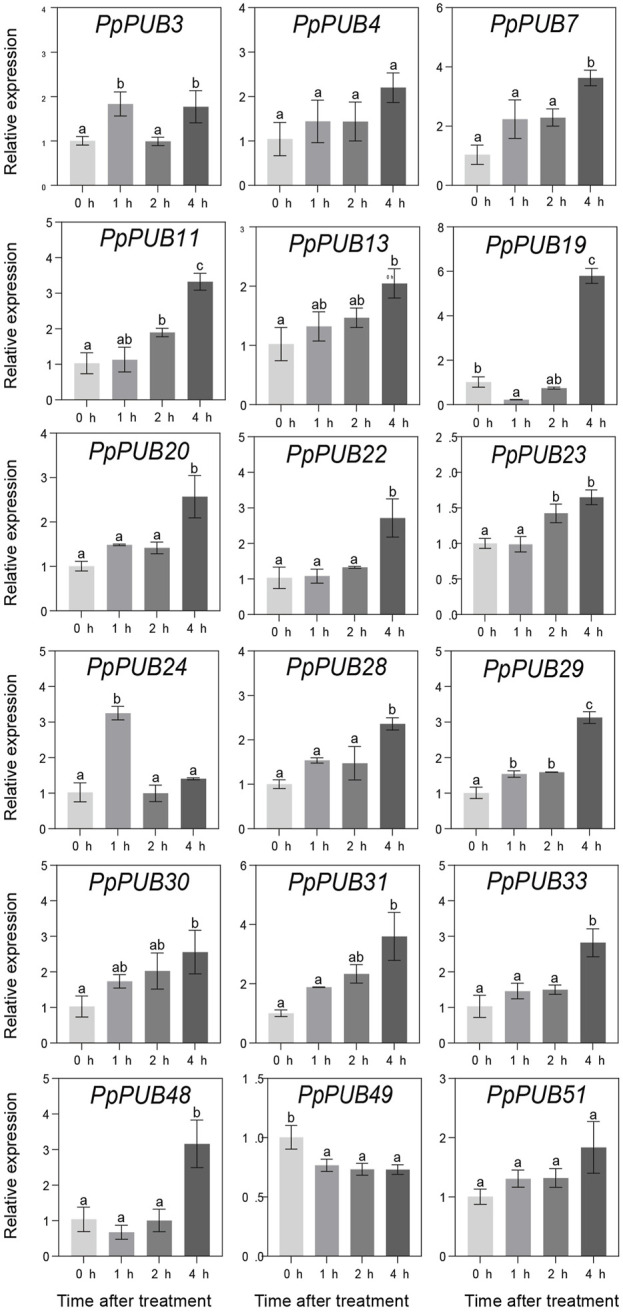
Relative expression levels of selected *PpPUB* genes under the exogenous ABA treatment. Total RNA was isolated from peach leaves after exogenous ABA treatment for 0 h, 1 h, 2 h, and 4 h, respectively. The extracted total RNA was then submitted to quantitative real-time PCR. Error bars represent the means ± standard deviation (SD) of three independent experiments. Significant differences were determined by Duncan’s multiple range test (p < 0.005; one-way ANOVA). The lowercase letters above each bar indicates significant differences. Bars with the same lowercase letter represent no significant difference, and *vice versa*.

**FIGURE 10:**
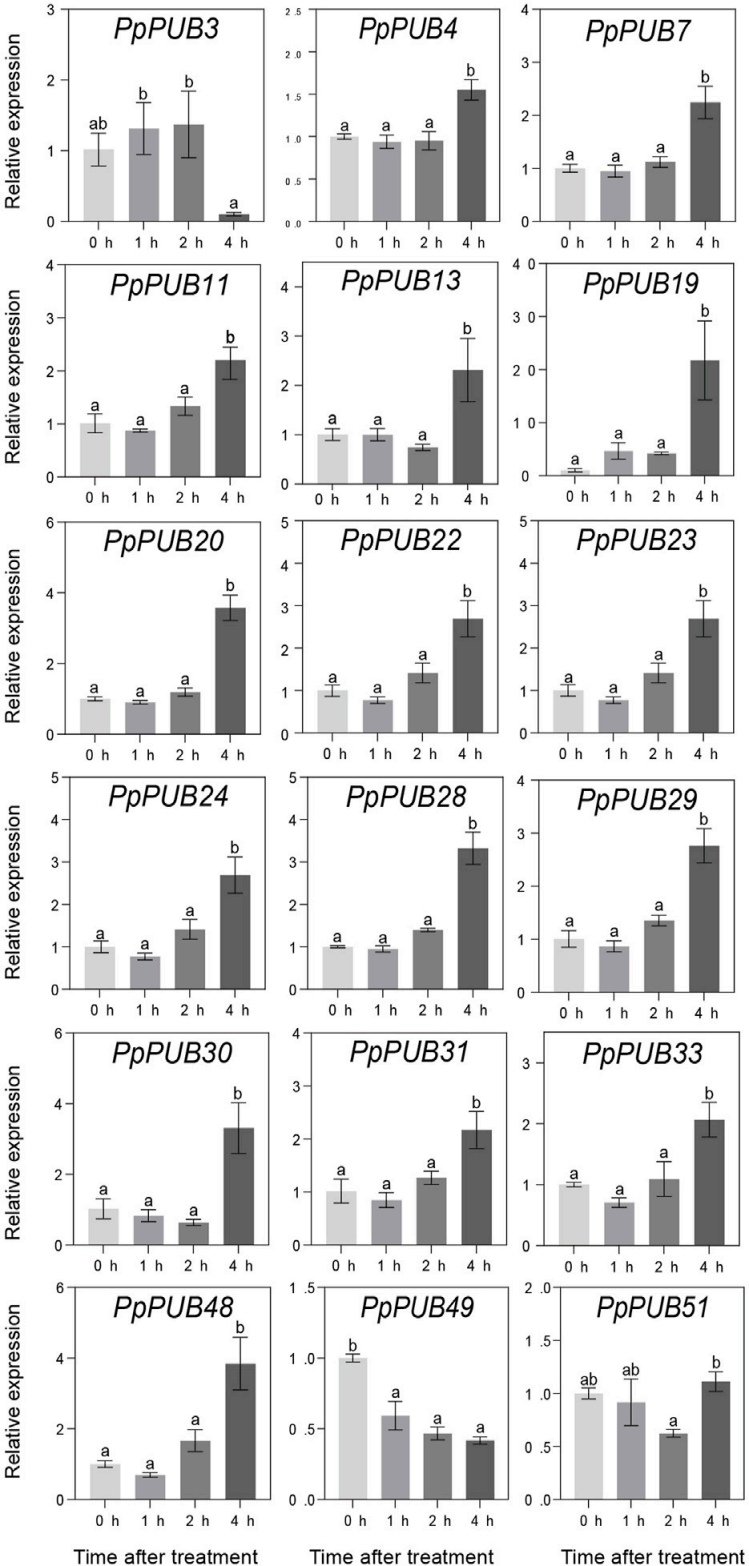
Relative expression levels of selected *PpPUB* genes under the exogenous GA3 treatment. Total RNA was isolated from peach leaves after exogenous GA3 treatment for 0 h, 1 h, 2 h, and 4 h, respectively. The extracted total RNA was then submitted to quantitative real-time PCR. Error bars represent the means ± standard deviation (SD) of three independent experiments. Significant differences were determined by Duncan’s multiple range test (p < 0.005; one-way ANOVA). The lowercase letters above each bar indicates significant differences. Bars with the same lowercase letter represent no significant difference, and *vice versa*.

**FIGURE 11:**
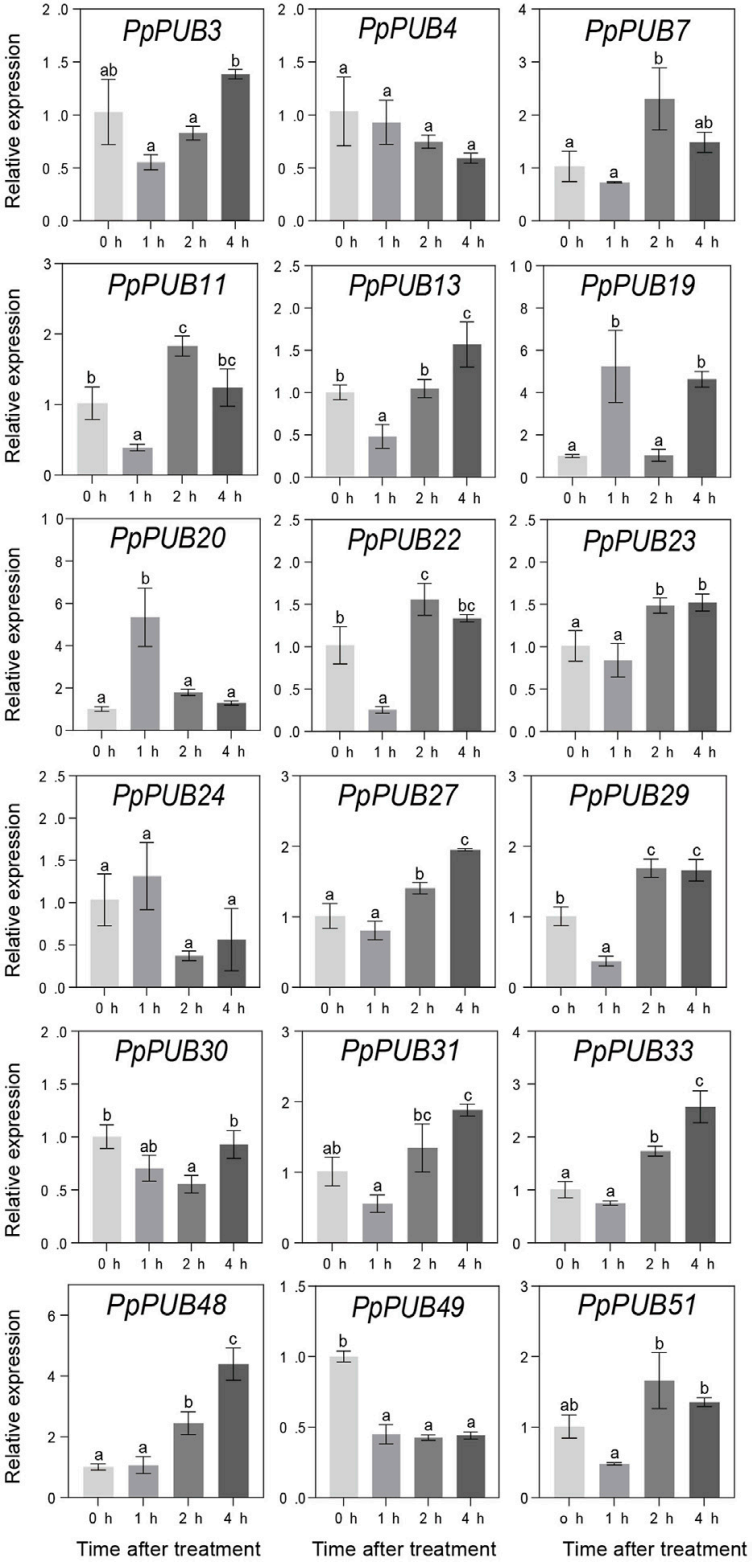
Relative expression levels of selected *PpPUB* genes under the exogenous IAA treatment. Total RNA was isolated from peach leaves after exogenous IAA treatment for 0 h, 1 h, 2 h, and 4 h, respectively. The extracted total RNA was then submitted to quantitative real-time PCR. Error bars represent the means ± standard deviation (SD) of three independent experiments. Significant differences were determined by Duncan’s multiple range test (p < 0.005; one-way ANOVA). The lowercase letters above each bar indicates significant differences. Bars with the same lowercase letter represent no significant difference, and *vice versa*.

**FIGURE 12:**
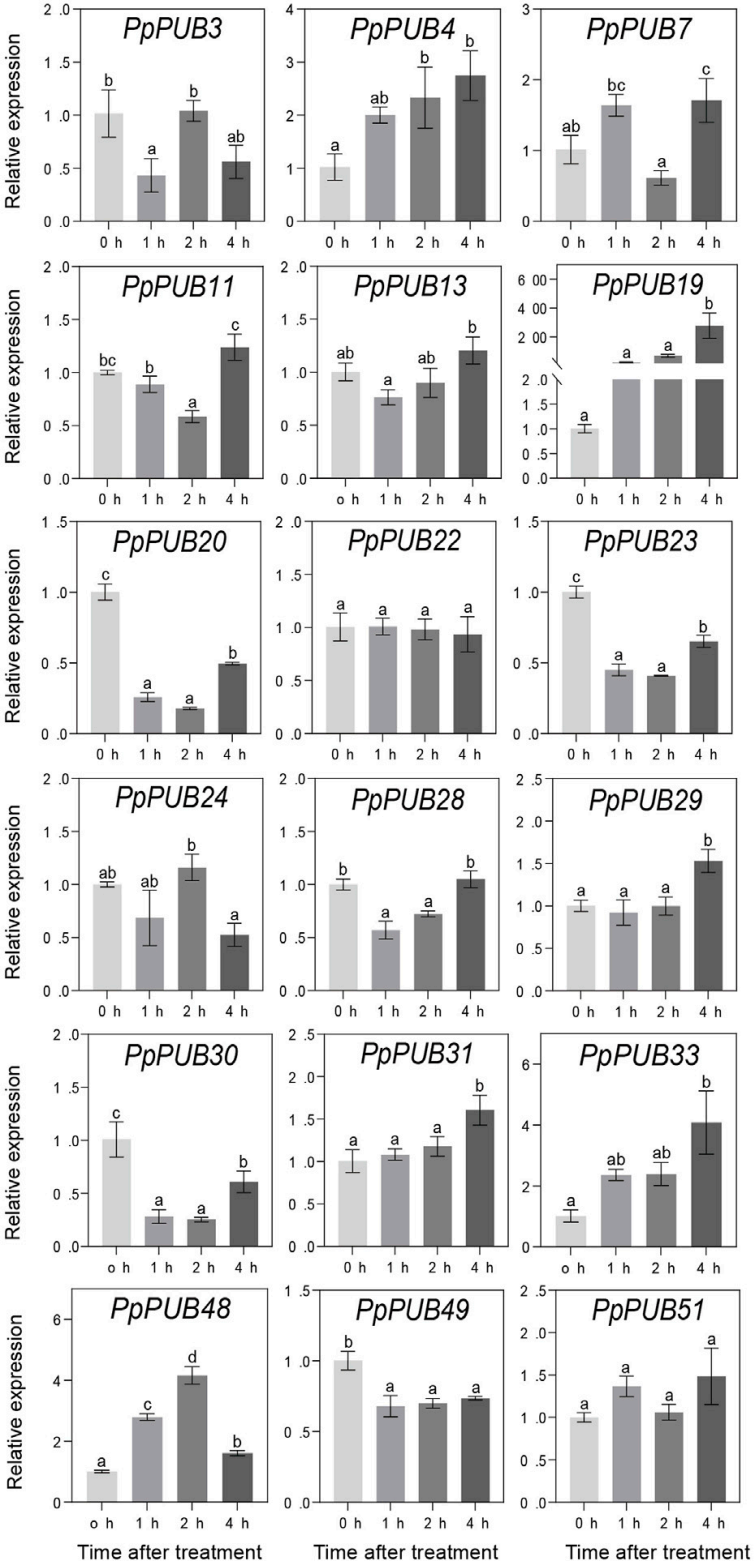
Relative expression levels of selected *PpPUB* genes under the exogenous 6-BA treatment. Total RNA was isolated from peach leaves after exogenous 6-BA treatment for 0 h, 1 h, 2 h, and 4 h, respectively. The extracted total RNA was then submitted to quantitative real-time PCR. Error bars represent the means ± standard deviation (SD) of three independent experiments. Significant differences were determined by Duncan’s multiple range test (p < 0.005; one-way ANOVA). The lowercase letters above each bar indicates significant differences. Bars with the same lowercase letter represent no significant difference, and *vice versa*.

ABA, GA3, IAA, and 6-BA are not only key regulators of fruit ripening but can also mediate the stress response of plants. This study evaluated the responsiveness of 18 representative genes in the *PpPUB* family to four hormone treatments. The results showed that, with ABA treatment, the expression of all 18 *PpPUB*, except *PpPUB49*, was upregulated (Figure 9). Among them, the transcription levels of *PpPUB19* were significantly downregulated within 2 h of ABA treatment and increased sharply after 4 h of ABA treatment (Figure 9). With GA3 treatment, the trend in expression of each gene was different, and the transcription levels of six *PpPUB* (*PpPUB7/11/22/23/29/51*) were downregulated after treatment and then peaked at 2 h (Figure 10). Notably, *PpPUB48* and *PpPUB49* were significantly upregulated and downregulated by GA3 treatment, respectively (Figure 10). With IAA treatment, the transcription levels of *PpPUB19* were significantly upregulated, and the transcription level of *PpPUB22* was almost unaffected (Figure 11). Notably, only the expression of *PpPUB49* was significantly inhibited by 6-BA treatment (Figure 12). These results indicate that hormones can induce the expression of *PpPUB* family genes.

### 3.8 Evaluation of salt tolerance of *PpPUB20* transgenic lines

**FIGURE 13:**
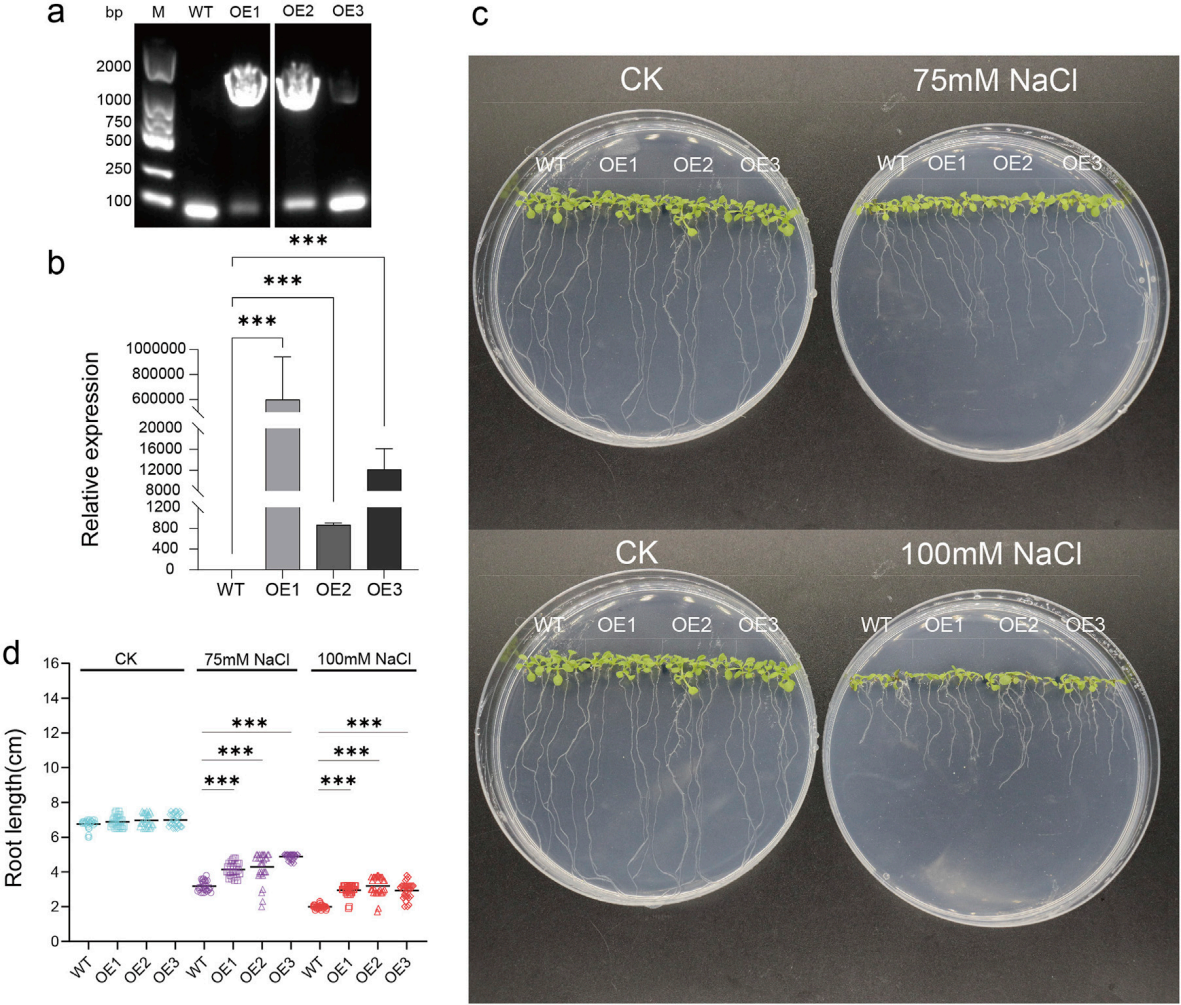
Salt-stress tolerance assay of transgenic Arabidopsis plants overexpressing *PpPUB20*. Salt tolerance assay of transgenic Arabidopsis plants overexpressing *PpPUB20*. **(a)** Detection of *PpPUB20* overexpression in Arabidopsis, PCR assay of genomic DNA of transgenic Arabidopsis; **(b)** relative expression of *PpPUB20* in control and Arabidopsis plants overexpressing *PpPUB20*, Error bars represent the means ± standard deviation (SD) of three independent experiments; **(c)** phenotypes of the transgenic seeds and WT after 10 days of salt stress; **(d)** WT after 10 days of salt stress, OE1, OE2 and OE3 root length comparison. An asterisk indicates that the value is significantly different from that of WT at the same time point (**P < 0.01; ***P < 0.001).

To further study the biological function of peach PUB genes under salt stress, based on the expression level after salt stress, *PpPUB20* was selected from 18 representative genes *Arabidopsis* col-0 plants (WT) were transformed by flower bud dipping method (Wiktorek-Smagur et al., 2009). Homozygous strains were assayed via conventional PCR and reverse transcription qPCR. Conventional PCR analysis showed that most strains were positive, and three overexpression (OE) lines, OE-1, OE-2, and OE-3, of *PpPUB20* were screened at the mRNA level (Figure 13a). The results of qPCR analysis showed that the abundance of *PpPUB20* transcripts in T3 transgenic lines (OE-1, OE-2, and OE-3) was higher than that in WT (Figure 13b). The WT and transgenic lines were cultured under normal conditions and salt stress environment (75 mM and 100 mM NaCl), respectively. Interestingly, under normal conditions, there was no difference in germination rate and phenotype between WT and transgenic lines. After 10 days of salt treatment, the three transgenic lines showed stronger tolerance to salt stress, and the root length was longer than that of WT plants (Figures 13c,d). The results showed that *PpPUB20* could improve the salt tolerance of *Arabidopsis* plants.

## 4 Discussion

The PUB genes belong to the E3 ubiquitin ligase family and are widely distributed in plants. Their characteristics and functions have been comprehensively studied in species such as *Arabidopsis* (Wiborg et al., 2008), tomatoes (*Solanum lycopersicum* L.) (Sharma and Taganna, 2020), and apples (*Malus pumila* Mill.) (Wang et al., 2020a). However, although peaches are one of the most popular economic fruit trees in China research on the members of the peach PUB family has been limited. In this study, we identified 51 *PpPUB* in the cultivated peach genome, which is fewer than reported in *Arabidopsis* (63), tomato (62), banana (*Musa nana* Lour.) (91) (Hu et al., 2018), pear (*Pyrus* spp) (62) (Wang et al., 2021), and peach (Tan et al., 2019) (54). The difference is perhaps because the screening conditions were more stringent and the different databases used as references. The 51 *PpPUB* were divided into six groups (Figure 1) according to the phylogenetic tree analysis, similar to that for white pear (*Pyrus bretschneideri* Rehder) of the same family (Wang et al., 2021). Most genes were located in the nucleus, chloroplasts, and cell wall (Table 1). The U-box domain is a functional domain in E3 ubiquitin ligases. In addition to the U-box structure, PpPUB contains several other domains, including Arm, WD40 repeats, the PLN03200 domain, and HEAT repeats (Figure 3). The domain distributions within the same group were more similar. It has been suggested that ARM duplication is the main factor mediating the interaction between U-box proteins and their substrates and ubiquitinates the substrates (Wang et al., 2020a). More importantly, the vast majority of U-box genes with biological functions are mainly derived from these U-box proteins with ARM repeats (Shu and Yang, 2017). In our study (Figure 3), all PpPUB proteins had U-box domain, and six members contained both U-box and ARM domains. Differentially distributed domains of the PpPUB family may have different biological functions.

In the analysis of cis-acting elements in the promoter, our results showed that multiple *PpPUB* contained cis-acting elements related to plant growth and development such as CAT-box and NON-box that specifically activate meristem in their promoters (Supplementary Table S2). These results revealed the putative involvement of PpPUB in peach growth and development, consistent with Saini’s report indicating that *AtPUB2* is involved in the regulation of *Arabidopsis* vegetative and reproductive growth (Lokesh et al., 2023). Of the 58 cis-acting elements, more than half (29) were related to the light reaction process, which may indicate a potential role for *PpPUB* in regulating plant photomorphogenesis (Supplementary Table S2). *Arabidopsis PUB13* can fine-tune photomorphogenesis and flowering time through *HFR1* (Li et al., 2012), whereas *AtPUB11* is regulated by photoperiod stimulation to regulate cell death (Liu et al., 2012). Among the cis-acting elements related to the hormone response, ABRE is the most abundant element in the *PpPUB* family and is related to ABA (Supplementary Table S2). U-box genes are involved in the regulation of ABA-mediated stress resistance in many plant species, including rice (Lv et al., 2022), poplar (*Populus* L.) (Tong et al., 2021), and wheat (Kim et al., 2022). In addition, the number of CGTCA and TGACG motifs involved in MeJA responsiveness was relatively high (Supplementary Table S2).

Among the cis-acting elements related to stress response in the *PpPUB* family are a large number of ARE, MBS, and LTR elements, which are related to anaerobic induction, drought response, and low-temperature response, respectively (Supplementary Table S2). Thus far, research on drought stress and low-temperature stress has been relatively comprehensive in many species (Min et al., 2016; Yao et al., 2017; Tong et al., 2021; Wang et al., 2021; Tang et al., 2022), but there are gaps in anaerobic induction-related research. There are three main ways to form a gene family: whole-genome duplication (WGD) or polyploidization, segmental duplication, and tandem duplication. The replication of chromosomal fragments leads to the replication of genes that are distant or located on different chromosomes. Tandem replication mainly occurs in chromosomal recombination regions, and members of the gene family formed by tandem replication are usually closely arranged on the same chromosome to form a gene cluster with similar sequences and functions (Richly et al., 2002; Leister, 2004). Our subsequent gene duplication and synchronic block analyses showed that dispersion was the main driving force for the formation of the peach *PUB* family (Supplementary Table S3). This duplication might be related to the evolution of repetitive sequences and species richness (Tachida, 1993; Xiong et al., 2020).

To further characterize the potential regulatory functions of *PpPUB* in peach growth and development, the expression patterns of 51 *PpPUB* were analyzed using RT-qPCR. Differences in the expression patterns of *PpPUB* family members in different tissues and organs may be related to their functional differences. Transcriptional analysis revealed that almost all *PpPUB* exhibited tissue- and organ-specific expression patterns. In addition, previous reports have shown that *PUB* respond to ABA signaling. In this study, ABA response elements were found in almost all putative promoter regions of the *PpPUB* (Supplementary Table S2; Figure 4). Consistent with this, the expression analysis of 18 representative *PpPUB* showed that exogenous ABA treatment significantly induced or inhibited several *PpPUB*, and the overall expression of 17 *PpPUB* increased after ABA treatment (Figure 9). These results are similar to those of a previous study (Jiang et al., 2023), indicating the potential involvement of *PpPUB* in the ABA signaling pathway. According to previous cis-acting element analysis results, it can be speculated that many *PpPUB* family genes are related to the gibberellin and auxin signaling pathways (Supplementary Table S2). Based on the quantitative results of the 18 representative *PpPUB* treated with GA3 and IAA in this study, almost all genes were significantly induced or inhibited by the treatments. In addition, we investigated the expression levels of 18 representative *PpPUB* under 6-BA treatment, and the results were similar to those of other hormone treatments. This suggests a potential role of *PpPUB* in peach growth and development. This is consistent with previous studies on the important role of U-box E3 ubiquitin ligases in plant growth and development (Mao et al., 2022). Therefore, it can be reasonably speculated that the potential involvement of *PpPUB* in peach growth and development and stress resistance may be related to the involvement of *PpPUB* in hormone signal transduction.

Abiotic stresses, including salinity and cold, are not conducive to plant growth and development, resulting in huge losses in peach yield (Lee et al., 2021). The ubiquitin-proteasome pathway is considered an important stress-response pathway. Many studies have shown that E3 ubiquitin ligase plays a role in the response to abiotic stress (Chen et al., 2021; Li et al., 2021; Mao et al., 2022; Tang et al., 2022). In this study, the differential expression of 18 representative *PpPUB* under salt stress and low-temperature conditions was investigated. The results showed that the expression of *PpPUB* began to differ 1 h after low-temperature treatment (Figure 8), and significant expression differences were observed after 2 h. Under low-temperature treatment, the expression of *PpPUB3/20/23/49* increased with an increase in the low-temperature treatment time, indicating that these genes may positively regulate the low-temperature response process. The changes in *PpPUB4/7/11/48* expression may indicate negative regulation of the low-temperature response process. In addition, among all *PpPUB* upregulated by low-temperature stress, *PpPUB20* showed the largest increase (Figure 8). Under salt treatment, *PpPUB19* was not expressed after 3 h treatment, which may indicate negative regulation of the salt-stress response process (Figure 7). *MdPUB29*, which is highly homologous to *AtPUB29*, may positively regulate salt tolerance (Han et al., 2019), suggesting that *PpPUB* in group 4, where *AtPUB29* was located, may also be involved in salt stress response. Salt stress may seriously affect the growth of plants. This study found that the expression levels of *PpPUB* (*PpPUB11/20/33/36*) in group 4 were upregulated after salt stress treatment (Figure 1). Interestingly, the expression level of *PpPUB20* was significantly upregulated under salt stress conditions. Therefore, it can be speculated that *PpPUB20* may play a crucial role in the salt and cold stress responses of peach trees. To further verify the role of *PpPUB20* in abiotic stress, it was heterologously overexpressed in *A*. *thaliana*. Under NaCl stress, *Arabidopsis* plants overexpressing *PpPUB20* had longer roots than those of WT plants (Figure 13c), indicating that the *PpPUB20* may positively regulate salt tolerance in *Arabidopsis* and confirming the conjecture of *PpPUB20* abiotic stress. However, the cellular mechanism of *PpPUB20* regulating salt stress response remains unclear and needs further study.

## 5 Conclusion

*PUB* play an important role in regulating plant growth and development and in response to various abiotic stresses. In this study, 51 *PpPUB* were identified and divided into six groups. Dispersion was the main driving force behind the formation of the peach *PUB* family. Repeated *PUB* may have undergone functional diversification because repeated gene pairs exhibited different expression patterns in different tissues and organs. Some *PpPUB* were found to be involved in the abiotic stress responses in plants. Our results on the functional identification of *PpPUB20* positively regulating the salt tolerance of *A*. *thaliana* lay a foundation for the functional study of *PUB* in peach in the future.

## Data Availability

The original contributions presented in the study are included in the article/Supplementary Material, further inquiries can be directed to the corresponding author/s.
